# Direct Observation of Thermal Hysteresis in the Molecular Dynamics of Barocaloric Neopentyl Glycol

**DOI:** 10.1021/acsaem.5c00495

**Published:** 2025-04-04

**Authors:** Frederic Rendell-Bhatti, Markus Appel, Connor S. Inglis, Melony Dilshad, Neha Mehta, Jonathan Radcliffe, Xavier Moya, Donald A. MacLaren, David Boldrin

**Affiliations:** 1 SUPA, School of Physics and Astronomy, University of Glasgow, Glasgow G12 8QQ, United Kingdom; 2 Institut Laue Langevin, 71 Avenue des Martyr, Grenoble 38000, France; 3 Department of Materials Science & Metallurgy, University of Cambridge, Cambridge CB3 0FS, United Kingdom; 4 School of Chemical Engineering, University of Birmingham, Birmingham B15 2TT, United Kingdom

**Keywords:** barocaloric, caloric, molecular crystal, plastic crystal, hydrogen bond network, quasielastic neutron scattering, neutron spectroscopy

## Abstract

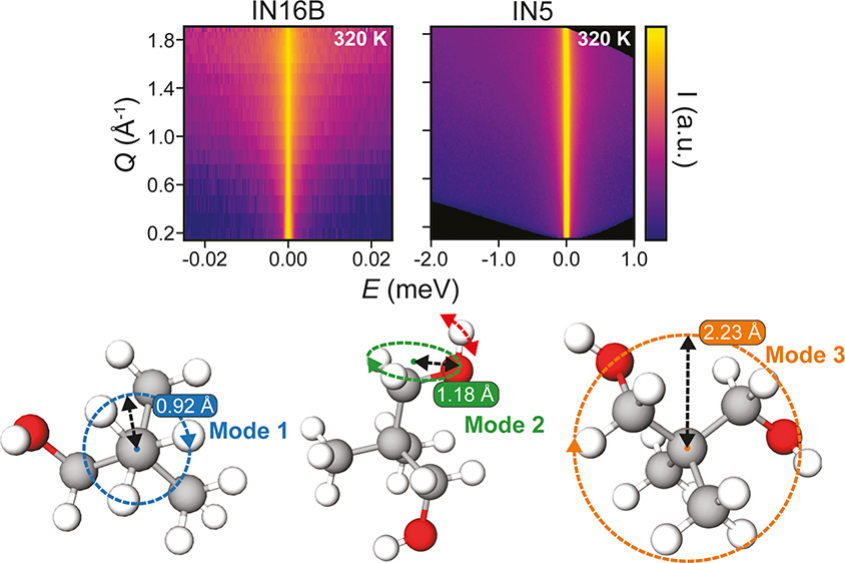

Barocalorics (BCs) are emerging as promising alternatives to vapor-phase refrigerants, which are problematic as they exacerbate climate change when they inevitably leak into the atmosphere. However, the commercialization of BC refrigerants is significantly hindered by hysteresis in the solid–solid phase transition that would be exploited in a refrigeration cycle. Here, we provide new insight into the hysteresis that is a critical step toward the rational design of viable BCs. By studying the benchmark BC plastic crystal, neopentyl glycol (NPG), we observe directly the liberation of the hydroxyl rotational modes that unlock the hydrogen bond network, distinguishing for the first time the molecular reorientation and hydroxymethyl rotational modes. We showcase the use of high-resolution inelastic fixed-window scans in combination with quasielastic neutron scattering measurements to build a comprehensive microscopic understanding of the NPG phase transition, directly tracking the molecular dynamics of the phase transition. Hysteresis previously observed in calorimetric studies of NPG is now observed directly as hysteresis in molecular rotational modes and hence in the formation and disruption of hydrogen bonding. Furthermore, by tracking the thermal activation of three main reorientation modes, we suggest that their fractional excitations may resolve an outstanding discrepancy between the measured and calculated entropy change. These results allow for a direct study of the molecular dynamics that govern the thermal hysteresis of small-molecule energy materials. They will be broadly applicable as many promising BC material families possess first-order transitions involving molecular reorientations.

## Introduction

Heat pumps, powered by renewable electricity, present a low carbon alternative for both heating^1^ and cooling applications.^2^ However, conventional heating, ventilation, and air conditioning (HVAC) systems employ gaseous refrigerants that leak into the atmosphere and have appreciable global warming potential (GWP).^3^ This problem will become a pressing concern as annual heat pump installations are projected to increase 11-fold by 2028 in the UK alone.^4^ As such, we require new refrigerant materials that are efficient, low cost, safe, and environmentally sustainable.^5^ Solid-state barocaloric (BC) materials exhibit significant latent heat associated with solid–solid (S–S) phase transitions that can be driven by pressure and therefore have the potential to revolutionize new and existing HVAC technologies through their use as working bodies.^6−10^ Being solids, these materials have negligible GWP and have the potential for even higher efficiency than vapor refrigerants.^11^ A promising class of BC materials includes the so-called molecular, or plastic, crystals (PCs), particularly neopentyl glycol (NPG), which exhibits a “colossal” BC effect.^12,13,17^ However, NPG is not itself viable for use in commercial HVAC systems. It exhibits substantial hysteresis when thermally cycled, necessitating high pressures to achieve the large reversible entropy changes needed for an efficient refrigeration cycle. Recent efforts have been made to both understand^14−17^ and influence^18,19^ the BC effect in NPG, which has become a benchmark for PC BCs. However, there is still a significant opportunity for the further development of PCs, which will rely on a better understanding of the microscopic mechanisms behind hysteresis in the S–S phase transition.

NPG, (CH_3_)_2_C(CH_2_OH)_2_, is a roughly spherical molecule consisting of two methyl (−CH_3_) and two hydroxymethyl (−CH_2_OH) groups that can rotate about bonds to a central carbon atom. Below its melting temperature, *T*_m_ = 400 K, NPG molecules adopt two distinct crystal structures. On heating at ambient pressures, NPG undergoes a S–S phase transition at *T*_0_ = 314 K, where hydrogen bonds between molecules are broken and the structure changes from a monoclinic ordered crystal (OC) phase to a face-centered-cubic (fcc) phase (see Supporting Information Figure S1). The latter is known as a disordered plastic crystal (PC) because molecular rotations are active and only the molecular center of mass has a well-defined location.^20,21^ The S–S, order–disorder phase transition is accompanied by large entropy (Δ*S* ∼390 J K^–1^ kg^–1^) and volume (Δ*V* = 5%) changes,^13^ and under adiabatic conditions, the latter allows material temperature changes to be driven by pressure, which is the BC effect. The phase transition is consistent with both the activation of rotational dynamic modes and a disruption of the hydrogen bond network that locks molecular orientations in the OC phase. The behavior of these rotational modes, and their thermal activation, offers direct insight into the mechanisms underpinning BC performance. Our goal is to use this insight into tailoring the microscopic mechanisms of the phase transition and thereby reducing thermal hysteresis during refrigeration cycles.

## Experimental Section

### Quasielastic Neutron Scattering (QENS) Data Acquisition

Powder samples of NPG (99% purity) were purchased from Sigma-Aldrich and used as received. Neutron scattering experiments were performed on the IN5 and IN16B spectrometers at Institut Laue-Langevin (ILL), France. IN5 is a disk chopper time-of-flight spectrometer, and IN16B is a backscattering spectrometer. On IN5, a wavelength of 6 Å was used, yielding an fwhm energy resolution of 60 μeV, dynamic range of ±1.3 meV, and *Q*-range of 0.10–1.89 Å^–1^ in this experiment. IN16B was used in standard configuration with a strained Si111 Doppler monochromator and analyzers, yielding an fwhm energy resolution of 0.75 μeV, dynamic range of ±0.028 meV, and *Q*-range of 0.19–1.89 Å^–1^. QENS measurements were obtained using scan times of 2 h on IN16B and 30 min on IN5. All QENS measurements were obtained on heating. FWS measurements were performed during a temperature ramp of approximately 0.5 K min^–1^ with alternating acquisitions of elastic (30 s) and inelastic intensity (90 s) at 3 μeV energy transfer. Approximately 0.5 g of sample was loaded into an aluminum can with annular geometry for measurements on both IN16B and IN5. Empty can and vanadium standard measurements were acquired using the same sample geometry and used to correct the NPG data. Data sets from each spectrometer were treated independently from each other using separate standard measurements for correction. We found that combining instrument QENS data sets (IN16B and IN5) gave the same results as treating the data sets separately. This is because the line widths of Modes 2 and 3 were within the instrument resolution of IN5 (making them undetectable) and the line width of Mode 1 was approximately equal to the energy range on IN16B, thus being well described by the flat background term. Resolution measurements were obtained at 2 K on each spectrometer, with the IN5 resolution having a systematic asymmetric and stepped profile intrinsic to the spectrometer.

### QENS Data Analysis and Fitting

All data were analyzed using Mantid v6.6.0 software. To calculate the EISF of each of the two modes above the phase transition observed on IN16B and IN5, we modified eq 4 to separate the slow and fast components. The EISF of the slow component was approximated according to
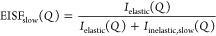


Likewise, we determined the EISF of the fast component according to
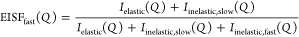
By including the intensity of the slow component in the numerator, it is equivalent to performing this measurement of the fast component on a spectrometer with an energy resolution of the slow component’s fwhm line width. This method of separating EISF components was used in previous studies of NPG^12^ and NH_4_BH_4_,^22^ where it successfully distinguished distinct rotational modes from within a single QENS signal.

Simultaneous global fitting of FWS and QENS data was performed to elucidate the behavior of the modes above the phase transition. Four data sets were included in the fit (IFWS at 3 μeV energy transfer, and three QENS scans (320, 330, and 340 K), each containing spectra for 16 different *Q* values. Equation 1 was used directly as the model function for QENS, representing an elastic peak, two Lorentzian components, and a flat background, all convoluted with the measured resolution function of the instrument. For the IFWS, eq 1 was modified by removing the elastic contribution and modeling a temperature dependence by choosing an Arrhenius law for the line widths, Γ_i_(*Q*):
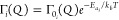


Subsequently, parameter ties were established between the different data sets to achieve the following:

- A global, *Q*-independent value for the activation energy of each process (*E*_*a*_1__and *E*_*a*_2__)
- Consistency between the IFWS line widths, calculated using the Arrhenius law above, and the fitted Lorentzian line widths Γ_i_(*Q*) from the QENS spectra at different temperatures
- Identical ratios of the amplitudes of Lorentzian contributions (*A*_1_(*Q*)/*A*_2_(*Q*)) across all spectra (QENS and IFWS) with the same *Q*

## Theory

QENS is used to measure inelastic cold neutron scattering from materials at low energy transfer of a few meV. Data sets appear as an elastic peak that is broadened by quasielastic scattered intensity due to energy exchange with rotational and translational diffusion processes within the sample.^23^ Thus, it is possible to use QENS to track low energy dynamic modes occurring within a sample by inspecting and characterizing the different quasielastic signals. The accessible time scales of these processes are primarily determined by the energy resolution of the spectrometer used to probe the sample, where better energy resolution enables the observation of processes with slower time scales (up to a few ns). Here, we utilized two spectrometers, IN16B and IN5 at Institut Laue-Langevin (ILL), in which each has a distinct energy resolution and dynamic range (see Experimental Section for instrument characteristics). This made it possible to access energy transfers from meV to μeV, corresponding to fast (picosecond) and slow (nanosecond) processes, respectively.

**Figure 1:**
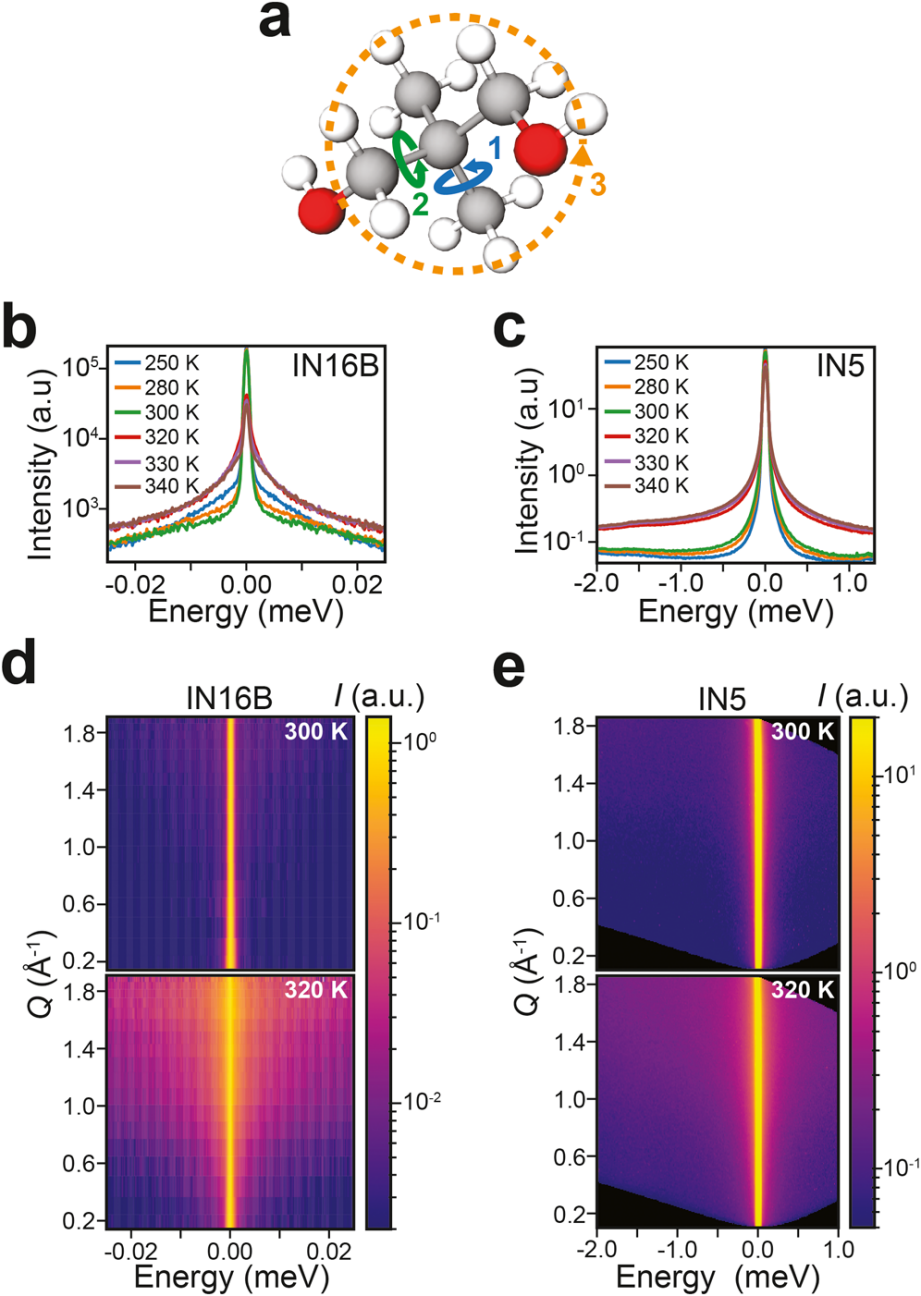
QENS data of NPG above and below its solid–solid phase transition. (a) Geometry of ^1^H dynamical modes in NPG. (b,c) QENS line profiles, i.e., *S*(ω) integrated over *Q* for each measured *T,* obtained from IN16B and IN5 instruments, respectively. Note the different energy axis scales. (d,e) *S*(*Q*,ω) maps below (300 K, top) and above (320 K, bottom) the phase transition, measured on IN16B and IN5 instruments, respectively.

The measurable quantity in a QENS experiment is the dynamic structure factor, *S*(*Q*,ω), which provides intensity as a function of the momentum (*Q*) and energy transfer (ω) of neutrons interacting with the sample. In NPG, scattering from hydrogen atoms (^1^H) dominates the QENS signal due to their large incoherent scattering cross section. *S*(*Q*,ω) can therefore be seen as a direct measurement of dynamic processes involving the motion of ^1^H. Based on the geometry of the NPG molecule and a previous theoretical study,^24^ we identify three accessible rotational modes in the PC phase. Mode 1 relates to the rotation of the methyl (−CH_3_) groups about the C–C bond axis. Mode 2 describes the similar rotation of hydroxymethyl (−CH_2_OH) groups about the C–C bond axis. Mode 3 is a more complicated rotational reorientation of the whole NPG molecule about its center of mass. In the OC phase, it is expected that only Mode 1 is accessible because the hydrogen bond network locks the hydroxyls and, hence, the entire molecule into a fixed orientation and position. These three rotational modes are shown schematically in Figure 1a. A previous QENS study of NPG^12^ was able to detect two of these three modes, where the third was not resolvable due to its slower time scale.^24^

The measured QENS data, *S*_measured_(*Q*,ω), obtained for NPG and shown in Figure 1d,e derives from a convolution of the instrumental resolution function, *R*(*Q*,ω), and the theoretical incoherent scattering function, *S*(*Q*,ω), through^25^
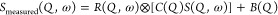
1where
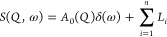
2and
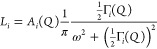
3

In eq 1, *C*(*Q*) is related to the Debye–Waller factor arising from the stochastic displacement of ^1^H from lattice vibrations. The effect of *C*(*Q*) will be omitted in the characterization of quasielastic processes; however, determination of the mean squared displacement (MSD) associated with this stochastic motion will be treated at the end of the Results and Discussion. *B*(*Q*) is a flat background term accounting for inelastic (high-energy transfer) contributions. All of the coherent and incoherent scattering arising from the sample is contained within *S*(*Q*,ω). In eq 2, the first term corresponds to the elastic component, modeled by a delta function, δ(ω), with amplitude *A*_0_; and the second term corresponds to the quasielastic component, modeled by a sum of Lorentzians (given by eq 3), with amplitudes *A*_*i*_, each corresponding to a distinct dynamical process with a characteristic line width (Γ_i_). The associated time scale, τ_i_, of each of these processes can be found through the relation . It should be noted that if Γ_i_ is much smaller than the width of the instrumental energy resolution, then the corresponding mode will contribute to the recorded elastic intensity, as if the associated rotation was stationary; in contrast, if Γ_*i*_ is substantially larger, then the corresponding mode will contribute to the measured background.

For NPG in its OC phase, we expect the measured QENS signal to resolve quasielastic contributions arising from Mode 1 only, so that eq 2 has only one Lorentzian term, while Modes 2 and 3 are also present in the PC phase, yielding three Lorentzian terms. As outlined below, Mode 1, the methyl group rotation, is typically modeled where ^1^H jumps between three equivalent sites equally spaced on a circle.^25^ Similarly, we will model mode 2 as a continuous rotation on a circle to track the hydroxyl ^1^H motion. Mode 3, relating to a full tumbling rotational motion of the NPG molecule, is modeled as continuous, isotropic rotation on a sphere. In each of these models, the ^1^H atoms hop instantaneously between equilibrium sites with a mean time between jumps given by τ_i_.

The amplitude of the elastic signal given by *A*_0_(*Q*) is known as the elastic incoherent structural factor (EISF), which is traditionally calculated as the measured proportion of elastic scattering within the total scattered intensity:
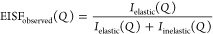
4When contributions from a single quasielastic component are considered, the EISF can be calculated simply using eq 4. However, if more modes are resolved, then a distinct EISF for each inelastic component may be determined^12,22^ (see the Experimental Section for details). The spatial geometry of the observed dynamic process leads to a characteristic functional form of EISF_observed_(*Q*), allowing it to be used to identify dynamics occurring within the sample. Finally, we can modify eq 4 to account for the possibility that only a fraction of scatterers participate in the observed process with a given geometry, modeled with EISF_model_(*Q*):
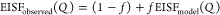
5where *f* is the fraction of scatterers mobile on the time scale of the spectrometer.^26−28^ In eqs 4 and 5, the nature of the *Q* dependence is directly related to the geometry of the motion of ^1^H. Thus, distinct geometric models (EISF_model_(*Q*)) can be used to fit the calculated EISF_observed_(*Q*) to determine the type of motion that produces the measured QENS signal.

## Results and Discussion

### QENS Fitting

Figure 1b,c shows the QENS data obtained from NPG on heating through its S–S phase transition (*T*_0_ = 314 K) from 250 to 340 K. The data are integrated across all measured *Q* for each spectrometer, with fwhm energy resolutions of 0.75 μeV for IN16B (Figure 1b) and 60 μeV for IN5 (Figure 1c). It is evident that both instruments detect a significant broadening of the elastic peak through the S–S phase transition and hence record an increase in the quasielastic signal that is made more apparent in the full *S*(*Q*,ω) data given in Figure 1d,e. This increase in signal is due to additional dynamical modes liberated in the PC phase. The IN5 data in Figure 1c show the phase transition as a jump in inelastic intensity between 300 and 320 K. Furthermore, these data visually show only a weak variation of broadening with *Q*, which suggests localized dynamics, as discussed later. After the phase transition occurs, there is a large increase in quasielastic signal across all measured energies. The data from IN16B in Figure 1b provide more details about the broadening at low energy transfers. Below the phase transition, we observe a decrease in quasielastic intensity within ±0.01 meV of the elastic peak from 250 to 300 K, suggesting that the frequency of this process increases with increasing temperature. Again, after the phase transition, we observe an increase in quasielastic signal with a weak dependence on *Q* now visible across the narrow energy range accessed by IN16B, shown in Figure 1d. The full energy range QENS data measured on IN5 can be found in Supporting Information Figure S2.

**Figure 2:**
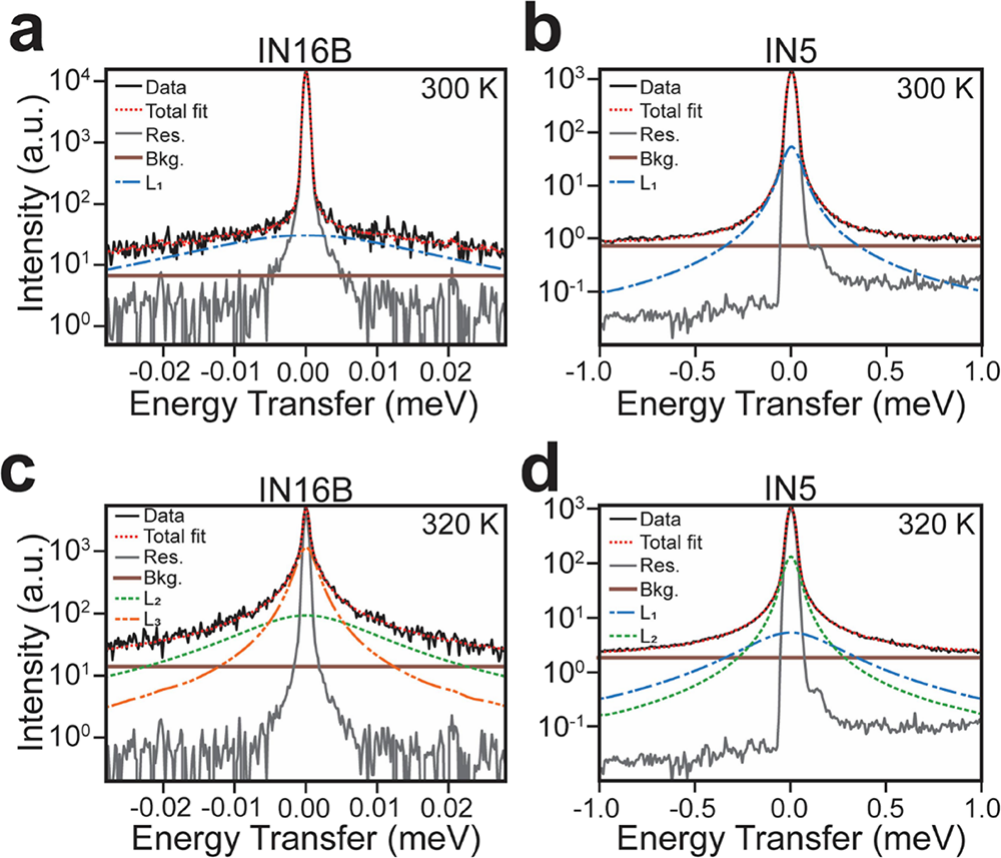
Representative QENS data fits for *Q* = 0.83 Å^–1^. (a,b) Example fits for IN16B and IN5 QENS data below the phase transition (300 K). Here, only a single Lorenztian is included in the model. (c,d) Example fits above the phase transition (320 K). In this case, two Lorenztians are required to fit the data. Simultaneous fitting across all *Q* was performed on each data set. The color and subscript of the Lorenztian fits are determined by the identification of the mode using EISF fitting in Figure 4. The asymmetric profile of the resolution function for IN5 in (b,d) arises from the spectrometer construction and is systematic across all data sets.

To determine the dynamic processes contributing to the quasielastic signal at each measured *T*, the full experimental data set was fitted using eqs 1 and 2. Fitting was performed simultaneously to each *Q* range within the data set, and example fits corresponding to *Q* = 0.83 Å^–1^, which is representative of the other *Q* values, are presented in Figure 2. In each case, it is apparent that there is a good fit to the data by using the model. Note that the colors of the Lorentzian components are chosen to specify distinct modes, as determined through *Q*-dependence of the EISF (eq 5), which will be presented below. The resolution function, *R*(ω), for each value of *Q* has been measured directly by recording a data set at 2 K. This is the reason for the gray traces in Figure 2 appearing noisy and also the reason for the asymmetric and stepped profile for the IN5 resolution function, which reveals some experimental artifacts at low signal levels that do not affect subsequent analysis.

We expect the mode identified at 300 K, i.e., below *T*_0_, to be the methyl group rotation. In the data from IN16B (Figure 2a), the fitted *L*_1_ term is very broad, approaching the limits of the instrumental dynamic range and so relating to a relatively high-frequency mode. In contrast, the same feature is detected on IN5 more clearly (Figure 2b). Figure 2c,d shows similar QENS data and fits above *T*_0_ at 320 K. For IN16B, in Figure 2c, the data are fitted well with two Lorentzian terms, one (*L*_2_) with a line width of the same order of magnitude as *L*_1_ in Figure 2a, while the other mode (*L*_3_) is significantly narrower. For IN5, in Figure 2d, the data are also fitted with two Lorentzians, but the line width of the first (*L*_1_) is orders of magnitude larger than that of either mode measured on IN16B at 320 K. Although the line width of the second component (*L*_2_) is comparable to that of the single mode (*L*_1_) observed at 300 K on IN5 in Figure 2b, it has been labeled as a distinct mode because the EISF data presented shortly shows distinct behavior (see Figure 4). These observations suggest that by considering the results from both spectrometers, we can observe at least three unique dynamic processes above *T*_0_.

**Figure 3:**
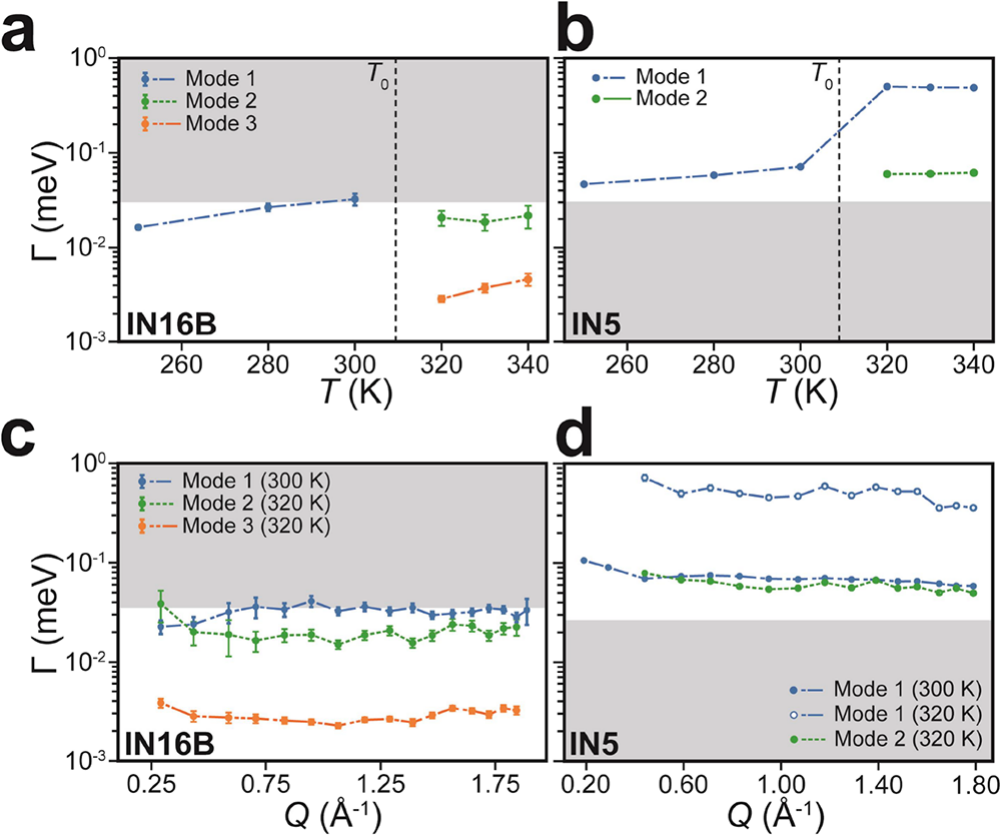
*T*- and *Q*-dependence of mode frequencies. (a,b) Γ(*T*) for each of the observed modes from the Lorentzian fits across the two instruments. The black dashed line indicates the equilibrium phase transition temperature. (c,d) Γ(*Q*) for each of the observed modes at temperatures just below (300 K) and above (320 K) the phase transition. Line color indicates the mode identity as found from fitting EISF (Figure 4). Gray regions represent the approximate time scales outside of the dynamic range of the spectrometer.

The *T-* and *Q*-dependences of the three modes are explored in more detail in Figure 3. Figure 3a,b plots the Γ_*i*_(*T*) of each mode across the two spectrometers, where the gray regions represent process time scales lying outside the dynamic range of the spectrometer. Below *T*_0_, we expect only the methyl group rotation to be active, and the only detectable Lorentzian contribution has a similar line width, Γ_1_ ≈ 50 μeV and τ_1_ ≈ 26 ps, measured across both spectrometers. Above *T*_0_, we observe two additional modes: Mode 2, with Γ_2_ ≈ 20 μeV and τ_2_ ≈ 66 ps, is resolved on both spectrometers, while Mode 3, with Γ_3_ ≈ 3 μeV and τ_3_ ≈ 440 ps, is observed only on IN16B. The original mode, retaining Γ_1_ ≈ 500 μeV, remains apparent in the IN5 data set but relates to a motion that has become too fast to be accessible to IN16B. This step-change in methyl rotation frequency across the phase transition has been observed previously^12^ and is likely due to different activation energies for the rotation in the PC phase compared to the OC phase because of the commensurate lattice expansion. Conversely, Mode 3 is not detected on IN5, as the line width is too small, and the time scale is too slow, for the motion to be resolved. Figure 3c,d then plots Γ(*Q*) at 300 and 320 K for IN16B and IN5, respectively. The trends are independent of *Q*, demonstrating that the modes are spatially localized and unlikely, for example, to represent diffusion processes.^29^

### Rotational Mode Identification

To identify the rotational processes that correspond to the modes shown in Figure 3, we determined the EISF associated with each Lorentzian. The calculated EISF data are then fitted using different geometric rotational models using their goodness of fit and molecular geometry considerations to assign each of the observed modes. We start by presenting the rotational models, with the assumption that the quasielastic signals are arising solely from incoherent ^1^H scattering due to jump diffusion between energetically equivalent sites with distinct τ_*i*_. The methyl rotation (Mode 1) is well described by the 120° three hop (3-Hop) model, which takes the following form:^25^
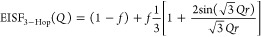
6where *r* is the rotational radius of the ^1^H motion associated with this mode. We identify Mode 2 with hydroxymethyl rotation, which should have a similar functional form to that of Mode 1, modeling the ^1^H scatterers as hopping between equivalent sites around a circle. However, recent molecular dynamics simulations^30^ have shown that because the hydroxymethyl rotation lacks 3-fold symmetry, it is expected that its motion is more complex (Supporting Information Figures S4 and S6), with more than three preferred orientations. We approximate this motion as hopping between six equivalent sites on a circle of radius *r*, as it has been shown that this is a good approximation for six or more hopping sites within the *Q*-range observed here.^25^ The EISF for this continuous hop (C-Hop) model is taken as^25^
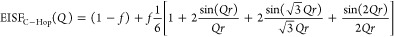
7

Finally, the EISF of Mode 3 is fitted assuming reorientation of the whole molecule using a model that describes ^1^H atoms continuously diffusing on a sphere with radius *r*:^25^
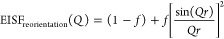
8

**Figure 4:**
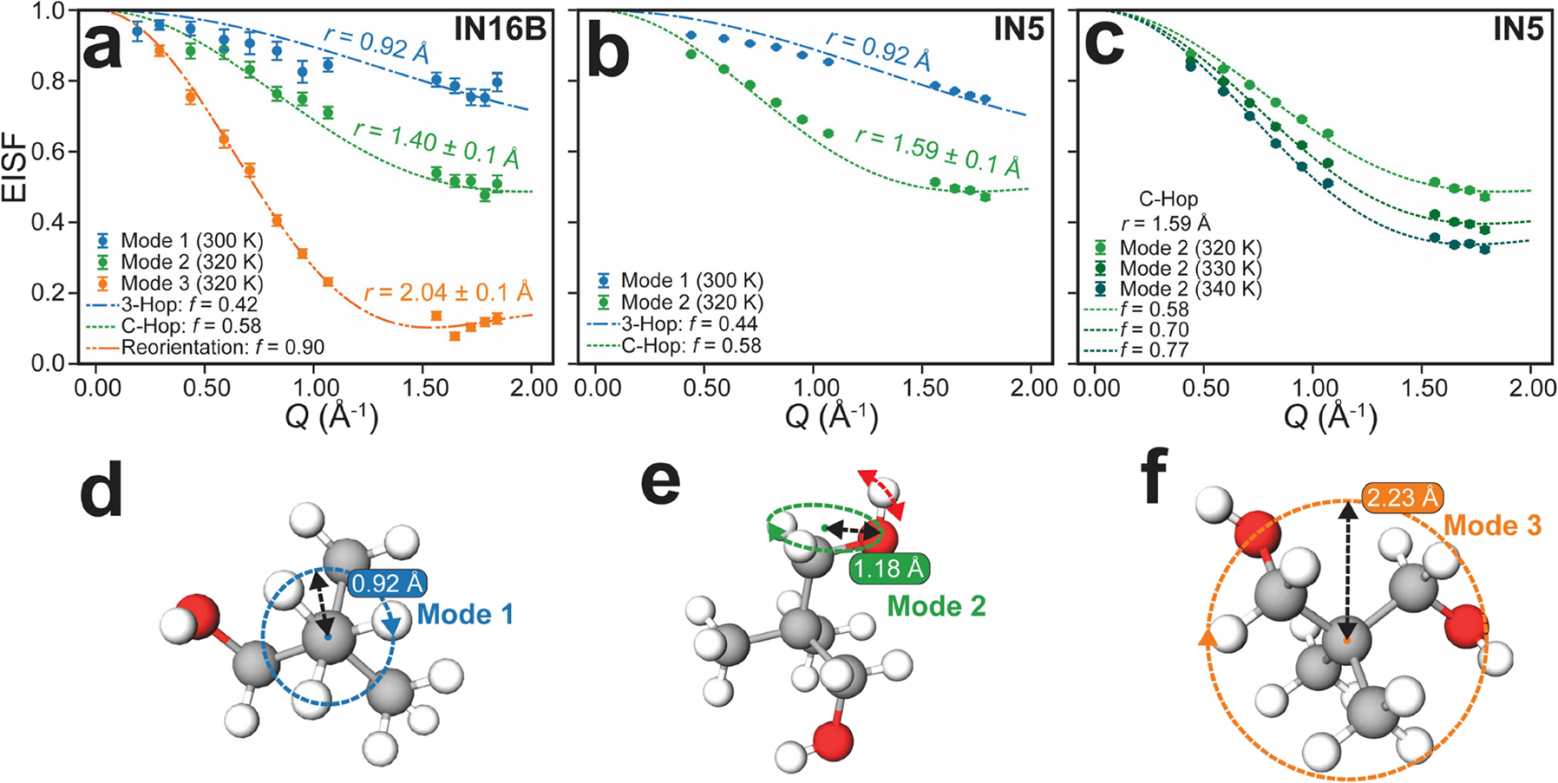
Fitting of EISF(*Q*) and identification of each of the observed modes. (a,b) Summary of EISF fitting for *T* = 300 and 320 K to data collected from (a) IN16B and (b) IN5. Here, three modes are identified and fit to incoherent scattering models as defined in the text. (c) *T*-dependence of the hydroxymethyl mode fraction fitted to IN5 data. (d–f) Schematics of the NPG molecule indicating the geometric origin for the radii of rotation obtained from fits in (a,b). Data for 1.1 < *Q* < 1.5 Å^–1^ was masked from fits due to the presence of Bragg peaks in this *Q*-range.

Figure 4 presents the EISF(*Q*) fits by using the above models for the observed rotational processes across both spectrometers. The distinct functional forms and fitted radii for EISF(*Q*) justify their identification with three distinct processes, each corresponding to a rotational mode with a unique geometry. First, inspecting the data from IN16B in Figure 4a, Mode 1 (blue circles) appears to be described well by the 3-Hop model defined in eq 6. By fixing *r* = 0.92 Å as calculated from NPG crystallography data,^31^ the best fit yields *f* = 0.42. Note that an upper bound of *f* = 0.5 is imposed for this fit since half of the scattering hydrogens are stationary on the time scale of IN16B in the OC phase and thus only contribute to elastic intensity. Next, the trend of Mode 2 (green circles) is described well by the C-Hop model defined in eq 7 using *r* = 1.40 ± 0.1 Å and *f* = 0.58, as determined by the best fit. This is slightly larger than expected from the geometry of the molecule, shown in Figure 4e. However, applying this model to the motion of the hydroxymethyl hydrogens is a substantial simplification because the hydroxymethyl group contains two distinct ^1^H types. Specifically, the hydroxyl ^1^H has an additional rotational degree of freedom about the C–O bond, indicated by the red arrow in Figure 4e, which is not captured by the model. While the model provides a good fit to our data and is consistent with expectations based on previous theory, further refinements to better capture hydroxymethyl rotation are expected. Finally, the trend of Mode 3 (orange circles) is described well by the reorientation model defined in eq 8 and yields a best fit rotational radius of *r* = 2.04 ± 0.1 Å and *f* = 0.90. This radius agrees well with the average hydrogen distance from the molecular center of mass if a full isotropic tumbling motion is considered, as indicated in Figure 4f. Our assignment of Mode 2 (τ_2_ ≈ 66 ps) as hydroxymethyl rotations and the slower Mode 3 (τ_3_ ≈ 440 ps) as molecular reorientations is consistent with recent molecular dynamics simulations on NPG.^30^ Here, the authors resolve the transitions between distinct molecular orientations but not the rotations of the hydroxymethyl group, as these are claimed to be occurring on a time scale shorter than the time interval used for configurational sampling. See the Supporting Information for calculations of the NPG geometry and justification for the radii used in the schematics of Figure 4d–f.

Moving on to the data collected for higher energy transfers, Figure 4b shows the corresponding data obtained from IN5. Immediately apparent is the insensitivity of IN5 to Mode 3, which is expected since Γ_3_ is smaller than the energy resolution of IN5. The fit for Mode 1, again fixing *r* = 0.92 Å, provides *f* = 0.44, which is very close to the value obtained on IN16B. For Mode 2, we again fit these data using the C-Hop model to represent the hydroxymethyl rotation yielding *r* = 1.59 ± 0.1 Å. This fit agrees well with the Mode 2 fit from the IN16B data in Figure 4a. We also note that the data presented in Figure 4b agrees well with previously obtained QENS data for NPG using a spectrometer with comparable energy resolution to IN5.^12^ That study utilized the neutron spectrometer AMATERAS with an energy resolution of 50 μeV, but the authors instead assigned these data to methyl rotation and molecular reorientation.

The Mode 2 fractional fits for both IN16B and IN5 seem to suggest that at 320 K, we detect 116% of the hydroxymethyl rotational motion with an expected upper limit of *f* = 0.5. This further indicates that the model is simplified for this mode. To determine how *f* varies with temperature on IN5, we fix *r* = 1.59 Å and refine *f*, the result of which can be seen in Figure 4c. Here, we observe a very clear trend of increasing *f* from *T* = 320 to 340 K. A possible explanation is that at higher temperatures, quasielastic intensity from the broadening reorientation motion is appearing in this model. The remaining EISF plots for other measured temperatures can be found in Supporting Information Figure S3.

### Fractional Fitting of Molecular Reorientation

**Figure 5:**
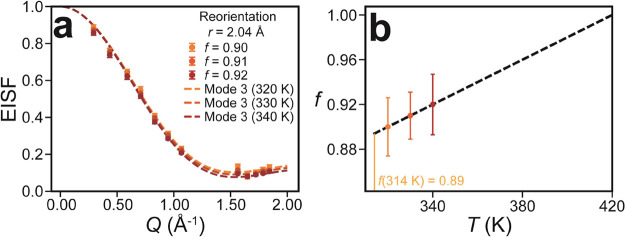
Reorientation mode fraction as a function of temperature. (a) EISF(*Q*) plots of the reorientation mode for temperatures above the phase transition. (b) Reorientation mode fraction, *f*, as a function of *T*; the line of best fit allows for the estimation of *f* = 0.89 at the phase transition, *T*_0_ = 314 K. This mode is fully liberated at 420 K, as determined by the best fit at *f* = 1.0.

The EISF analysis presented in Figure 4 highlights the importance of the fractional fitting parameter, *f*, to provide meaningful fits to all of the observed modes. The inclusion of this parameter has been used extensively to study other material systems,^26−28,32^ where it is often interpreted as representing a fraction of “bound” or otherwise “noncontributing” scatterers. Here, we offer possible physical interpretations of this parameter in the fitting of the isotropic molecular reorientation (Mode 3) in NPG using the high-resolution IN16B data shown in Figure 5a. The motion has been previously described as a “complex reorientation mode” with a nontrivial energy landscape, consisting of rotation about an axis that is also slowly rotating.^12,24^ The characteristic time scale of Γ_3_ is ∼650 ps at 320 K, much faster than the time-resolution limit of IN16B (∼4 ns). Nevertheless, Figure 5a suggests that the fraction of observed scatterers using IN16B increases with increasing *T*. By extrapolating this trend, in Figure 5b, we assess how the scatterers contribute to the mode at *T*_0_ and beyond. It is a subtle effect, but the fitted fraction, *f*, increases from 0.89 to 1.00 between 314 and 420 K. The latter temperature is close to the melting temperature of NPG (402 K), where the molecules obtain additional translational degrees of freedom. This observation indicates that (1) not all the NPG molecules are freely rotating immediately after the phase transition and/or (2) not all orientations are equally accessible to the molecules at the phase transition. The first possibility could be attributed to structural inhomogeneities in the material, for example, grain boundaries recently characterized using polarized light^15^ and electron^16^ microscopy, that result in different local environments and geometric hindrance of the molecules. Another explanation for the first possibility is that at lower temperatures, transient hydrogen bonds lock a small fraction of molecules in place for some time. The second possibility agrees with previous theoretical work that showed that the activation energy barrier for molecular reorientation is not isotropic.^24^

Our observation that the rotational motion of NPG molecules is not fully liberated at *T*_0_ has implications for efficient operation of NPG as a BC. Operating very close to T_0_ at around 320 K (see Supporting Information Figure S1 and ref (33)) will not maximize the entropy changes associated with the phase transition. It is also important to estimate configurational entropy changes around the phase transition. Such calculations allow an estimation of the BC performance of PCs but can greatly overestimate^24^ or underestimate^34^ the entropy changes obtained from calorimetry measurements. A more gradual liberation of molecular reorientations may explain why the calculated configurational entropy change, Δ*S* = *R* ln(180) = 415 J K^–1^ kg^–1^, is overestimated by around 6%. Taking instead the configurational entropy change to be Δ*S* = *R* ln(180 × 0.89), essentially reducing the number of accessible microstates at *T*_0_, yields a value of 405 J K^–1^ kg^–1^, closer to the experimentally determined value of ∼390 J K^–1^ kg^–1^^12,33,35−37^ and consistent with the value of 400 J K^–1^ kg^–1^ recently obtained from the molecular dynamics simulations of NPG.^30^ Of course, this estimation only considers the configurational entropy of the molecules and does not include other minor contributions such as lattice distortions. Regardless of the true physical interpretation of *f*, this study indicates that configurational entropy change calculations should not be solely relied on for estimating the BC performance in candidate materials.

### Fixed Window Scans

**Figure 6:**
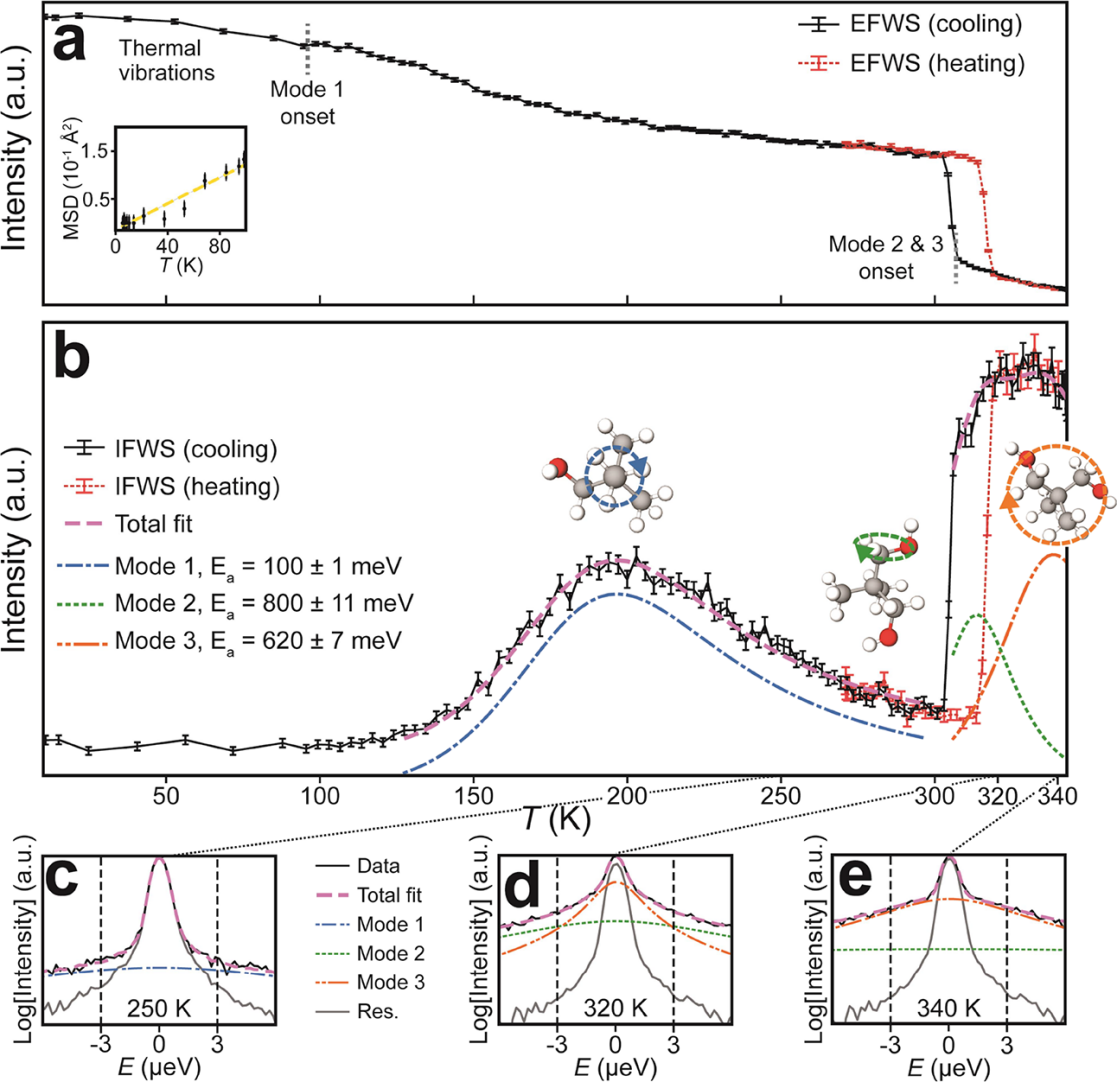
Combined FWS and QENS global fitting analysis**.** (a) EFWS (0.0 μeV) for NPG on heating and cooling. Inset: MSD(*T*) obtained from fitting of EFWS for *T* < 100 K. (b) IFWS (±3.0 μeV) for NPG on heating and cooling. Fittings of IFWS peaks on cooling at 200, 310, and 340 K correspond to methyl, hydroxymethyl, and molecule reorientations, respectively, as shown by the inset schematics. Global fits including QENS scans shown in (c–e) provide measured activation energies of 100, 800, and 620 meV, respectively. All data shown is for *Q* = 0.83 Å^–1^, representative of other *Q* values. See the Experimental Section for fitting details. The width of the energy window for both EFWS and IFWS is ±0.75 μeV.

Finally, we turn our attention to fixed window scans (FWSs) using the IN16B instrument. This unique technique allows the QENS signal to be monitored at specific energy transfers during the thermal processing of a sample. Figure 6a shows the elastic FWS (EFWS), and Figure 6b shows the inelastic FWS (IFWS) for NPG on heating from 280 to 345 K (red data) and cooling from 345 to 10 K (black data) for *Q* = 0.83 Å^–1^. The elastic signal corresponds to the scattered intensity about zero energy transfer within instrumental energy resolution (±0.75 μeV). Similarly, the IFWS was collected with the Doppler monochromator set up to collect inelastic signals corresponding to an energy transfer of ±3 μeV. The two data sets complement one another. A loss of EFWS intensity is accompanied by an increase in inelastic scattering, and so, the general trend in Figure 6a shows gradual thermal activation of vibrational and rotational modes in NPG. However, because the IFWS records only a narrow window, it does not simply show the inverse of the EFWS, and the energy window can be selected to accentuate differences in the QENS peak broadening. Here, the window chosen focuses on the clear differences in the peak shoulder observed between 250 and 320 K in Figure 1b, where the intensity appears to first drop for temperatures below *T*_0_ and then suddenly increase above *T*_0_.

Between 10 and 100 K, there is little IFWS signal in Figure 6b, since any rotational motion occurs on a time scale too slow to be observed. The simultaneous slow reduction in EFWS (Figure 6a) arises from thermal vibrations and librations associated with the ^1^H atoms. This temperature region was fitted across all *Q* to obtain the Debye–Waller factor mean squared displacement (MSD) associated with the low-*T* thermal motion, as shown in the inset of Figure 6a. The linear best-fit to this data provides the temperature-dependence of this motion as ∼1.4 × 10^–3^ Å^2^ K^–1^, which tentatively equates to an MSD at 300 K of 0.4 Å^2^. At 100 K, the inelastic signal in Figure 6b increases as the elastic signal in Figure 6a starts to decrease due to the appearance of the methyl group rotation (Mode 1). This gradual loss of elastic intensity continues across the 100–300 K range, yielding a distinct peak in the IFWS signal in Figure 6b. This peak can be attributed to the *L*_1_ term “moving through” the fixed energy window as the associated mode decreases in frequency with decreasing temperature. By fitting the thermal variation of the *L*_1_ term (blue line) and including a flat background (not shown for visual clarity), as described previously and assuming an Arrhenius behavior,^38^ we obtain an activation energy for Mode 1 of 100 ± 1 meV. Figure 6c shows the QENS scan obtained at 250 K and demonstrates how the IFWS signal is obtained at the black dashed lines located at ±3 μeV.

Both FWS plots in Figure 6a,b show a clear hysteresis in the modes detected by inelastic neutron scattering. At ∼314 K on heating, there is a large discontinuous decrease in elastic signal in Figure 6a, consistent with the phase transition temperatures determined by calorimetry (Supporting Information Figure S1), corresponding to the increase in inelastic scattering through the S–S phase transition. A similar abrupt increase in the elastic signal occurs instead at ∼304 K on cooling in Figure 6a, corresponding to the S–S phase transition driven by the eventual formation of the hydrogen bond network. The step-change in inelastic signal in Figure 6b highlights the fact that on heating (cooling), Modes 2 and 3 disappear (appear) on the breaking (formation) of the hydrogen bond network. In NPG, the appearance of these modes implies the loss of the hydrogen bond network that is mediated by the hydroxyls; hydroxymethyl rotation and full molecular reorientation can only occur when the hydrogen bond network is lost.

On cooling from 343 to 304 K, the IFWS intensity passes through a maximum at ∼320 K, which we attribute to the *L*_2_, *L*_3_ terms narrowing, demonstrated by the QENS data in Figure 6d,e. This reduction in the frequency of Modes 2 and 3 occurs on cooling until a temperature of ∼304 K, where the *L*_2_, *L*_3_ terms vanish, the tumbling motion and hydroxymethyl rotation both stop, and the molecules are locked back into a hydrogen-bonded lattice. With an energy offset of 3 μeV, the IFWS data above 304 K in Figure 6b contain signals from both *L*_2_ and *L*_3_ terms. This can be confirmed with the QENS scans in Figure 6d,e at 320 and 340 K, respectively, where *L*_2_ (green line) and *L*_3_ (orange line) intercept the dashed lines at ±3 μeV. Using this observation, we combine the IFWS data with the QENS scans at 320, 330, and 340 K in a global fit to characterize the behavior above the S–S phase transition across the full *Q*-range (see the Experimental Section for fitting details). Additional IFWS and QENS fits for low- and high-*Q* values can be seen in Supporting Information Figure S7. This fitting procedure yields activation energies of 800 ± 11 meV for Mode 2 and 620 ± 7 meV for Mode 3. The relative magnitudes of these values match those calculated by others from simulations^24^ and suggest that, surprisingly, the hydroxymethyl rotation has a higher activation energy than the molecular reorientation in the PC phase.

Our global fitting procedure allows us to extract the individual behaviors of the hydroxymethyl rotation and molecular reorientation in the hysteresis region (304–314 K). Specifically, on cooling, the line width of the *L*_3_ term (orange line) narrows to below the 3 μeV energy window offset, approaching the energy resolution of the spectrometer. This indicates that the molecular reorientations continue to lower temperatures than during the heating cycle but cease as the hydrogen bond network is formed. Conversely, the line width of the *L*_2_ term (green line) contributes almost all the IFWS signal below ∼310 K, indicating that hydroxymethyl rotations are more active despite the significant slowing of whole molecule rotations.

### Discussion

Our results demonstrate that IFWS and EFWS techniques provide a direct experimental method for probing orientational degrees of freedom in molecular crystals, closely related to the presence of hydrogen bond networks. Similar recent studies are often carried out using less direct techniques such as Raman spectroscopy,^24,39−41^ calorimetry,^13,33,42,43^ or QENS analysis at a small number of discrete temperatures.^12,28,34,44^ Here, we are able to measure the material behavior continuously through thermal cycles, giving direct observation of molecular dynamics within hysteretic regions of a phase transition while simultaneously extracting key physical parameters such as activation energies.

For the case of NPG derivatives and related PCs, it is clear that supercooling and hysteresis effects are dependent on the establishment of a hydrogen bond network. Although hysteresis effects can be expected within a first-order order–disorder phase transition, there is scope to reduce that hysteresis and the associated stochastic supercooling effects and thereby improve the viability of NPG as a commercial refrigerant. In the case of NPG, the entropic and enthalpic changes exploited by a refrigeration cycle are derived from the onset of full rotational disorder of the NPG molecules. There is, however, no *a priori* reason to assume that full molecular rotation should be commensurate with activation of the hydroxymethyl rotation since the energy landscapes for these two excitations are different. Nevertheless, we demonstrate here that the latent heat associated with the phase transition is unlocked by activation of the hydroxymethyl rotational mode. Once active, full molecular reorientation is facile because the hydrogen bond network is simultaneously disrupted, and the thermodynamic barriers to molecular reorientation are smaller than those that constrain the hydroxymethyl group. Evidence for this is already hinted at through (a) the drastic increase in transition temperature with increased number of hydroxymethyl groups in the wider group of hydroxylated neopentane PCs and (b) theoretical modeling of NPG showing that molecular reorientations are of lower energy than hydroxymethyl rotations.^24^ Our results suggest a route for intrinsic control of hysteresis through modification of the energy landscape for hydroxymethyl rotation, for example, short-range disruption of the hydrogen bonds through blending plastic crystals with different numbers of hydroxymethyl, amino, or other polarized functional groups capable of participating in hydrogen bonding. We propose the introduction of a fraction of molecules into the hydrogen bonded network that have lower barriers to rotation. These could provide the nucleation events required to suppress the hysteric supercooling effects that are the only practical hurdle in the development of barocaloric refrigeration devices.

## Conclusions

By considering data from two complementary neutron spectrometers (IN5 and IN16B), we have directly tracked the molecular dynamics underpinning the barocaloric performance in the benchmark barocaloric material, NPG. Utilizing the increased energy range of the complementary spectrometers, we experimentally resolve for the first time the three rotational modes of NPG: methyl, hydroxymethyl, and molecular reorientation. A clear link is observed between the disruption of the hydrogen bond network and the solid–solid phase transition, in good agreement with previous studies on NPG. The supercooling effect, where the “freezing” transition occurs at a lower temperature than “melting”, arises because residual hydroxymethyl rotations suppress hydrogen bonding. Thus, modification of the energy landscape associated with hydroxymethyl rotations may provide opportunities for engineering reduced thermal hysteresis in future materials. Our analysis permits an estimate of the fraction of molecules actively reorienting as a function of temperature, providing insight into previous discrepancies between experimental and theoretical configurational entropy change calculations. Global fitting of combined high-resolution inelastic fixed window scans and QENS measurements enables detailed insight into the temperature dependence of the detected modes from 10 K to well above the solid–solid phase transition. This allows for the extraction of mode activation energies that are in good agreement with previous theoretical work. Within the field of barocalorics, these results provide a deeper understanding of the detrimental hysteresis of the first-order barocaloric phase transition in NPG. This insight can be exploited to tailor the microscopic mechanisms of the phase transition in barocaloric molecular crystals toward reducing thermal hysteresis during heating or cooling cycles. More generally, our study demonstrates the power of using a combined inelastic fixed window and quasielastic neutron scattering approach to build a more complete understanding of the molecular dynamics that drive the phase transitions and thermal hysteresis critical to the functionality of many first-order phase transition materials.

## Supporting Information Available

NPG calorimetry data; full energy range IN5 data; additional EISF data; NPG molecular geometry; additional IFWS fits (PDF)
