# Supplementary material for: Direct Observation of Thermal Hysteresis in the Molecular Dynamics of Barocaloric Neopentyl Glycol
Source: ACS Appl Energy Mater. 2025 Apr 4;8(7):4793–802. doi: 10.1021/acsaem.5c00495 (PMC12001246; doi:10.1021/acsaem.5c00495)
Supplement: Supplementary file 1 — ae5c00495_si_001.pdf [file ae5c00495_si_001.pdf]

Supporting Information:

Direct observation of thermal hysteresis in the molecular dynamics of barocaloric neopentyl glycol

Frederic Rendell-Bhatti[1], Markus Appel[2], Connor S. Inglis[1], Melony Dilshad[3], Neha Mehta[4], Jonathan Radcliffe[4], Xavier Moya[3], Donald A. MacLaren[1] and David Boldrin[1]*

[1]SUPA, School of Physics and Astronomy, University of Glasgow, Glasgow G12 8QQ, United Kingdom

[2]Institut Laue Langevin, 71 Avenue des Martyr, 38000 Grenoble, France

[3]Department of Materials Science & Metallurgy, University of Cambridge, Cambridge, CB3 0FS, United Kingdom

[4]School of Chemical Engineering, University of Birmingham, Birmingham B15 2TT, United Kingdom

**Corresponding author email: fred.rendell@glasgow.ac.uk*

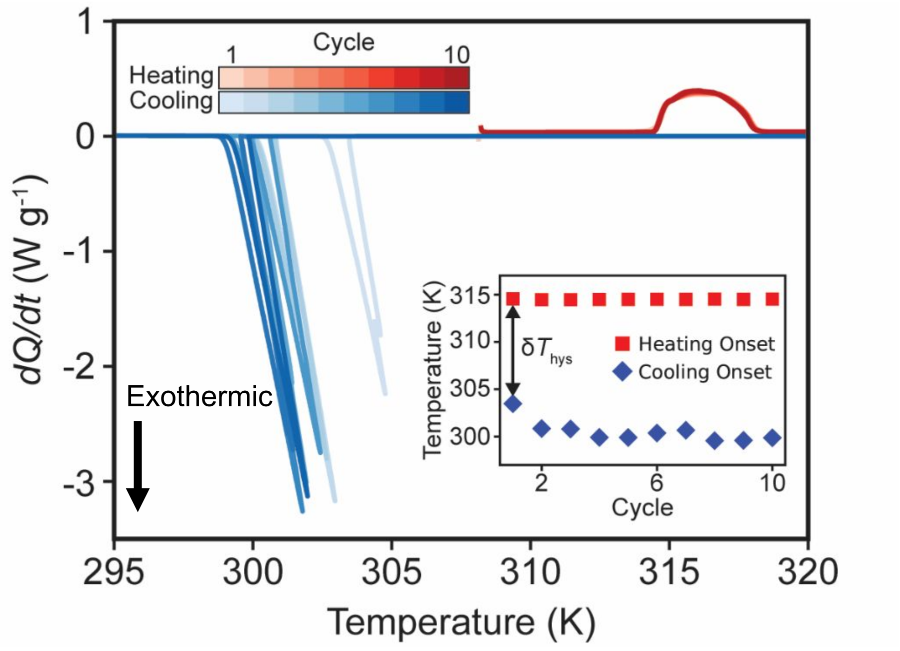

Figure S1 | Ambient pressure differential scanning calorimetry data for NPG. 10 successive heating and cooling cycles of NPG, showing thermal hysteresis and a heating transition of T_{0_start} = 314 K and T_{0_end} = 318 K. On heating, below 314 K NPG exists in an ordered crystal phase, above 318 K it exists in a plastic crystal phase. Scanning rate 0.5 K min^{-1}. This data has been published by us previously [1]. Asymmetry observed in the exothermic peaks is due to supercooling effects.

Figure S1 shows ten successive heating and cooling cycles of neopentyl glycol (NPG) using ambient pressure differential scanning calorimetry (DSC), indicating a clear thermal hysteresis between heating and cooling phase transitions. Furthermore, the temperature span of the heating transition is around 4 K, from T_{0_start} = 314 K to T_{0_end} = 318 K. The reorientation mode provided in Fig. 5 in the main manuscript indicates that this mode is not fully liberated (f = 1) until 357 K, 39 K above the endpoint of the phase transition as detected through latent heat in Fig. S1. More information Regarding the above data can be found in Ref. [1] .

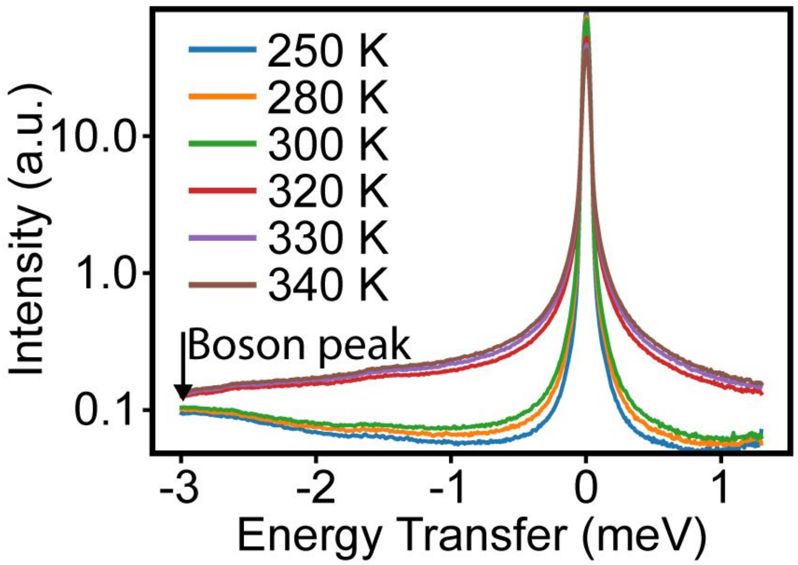

Figure S2 | QENS data summed for 0.10 < *Q* < 1.89 Å^{-1}, obtained from IN5. Full energy range QENS data obtained from IN5 showing the Boson peak around -3 meV in the OC phase of NPG at 250 K, 280 K and 300 K.

Figure S2 provides the full energy range of the acquired data for NPG on the neutron spectrometer IN5. This data shows the appearance of a so-called “boson peak”, common in orientationally disordered materials[2,3], at around -3 meV in the ordered crystal phase only, at temperatures of 250 K, 280 K and 300 K. The exact origin of this peak is unknown; however, it is accepted that it arises from intermediate-range order[4]. This work was focused on understanding the very short-range dynamics of NPG, and as such we limited the energy range to ± 1 meV in the main manuscript, to avoid fitting the boson peak or other inelastic processes.

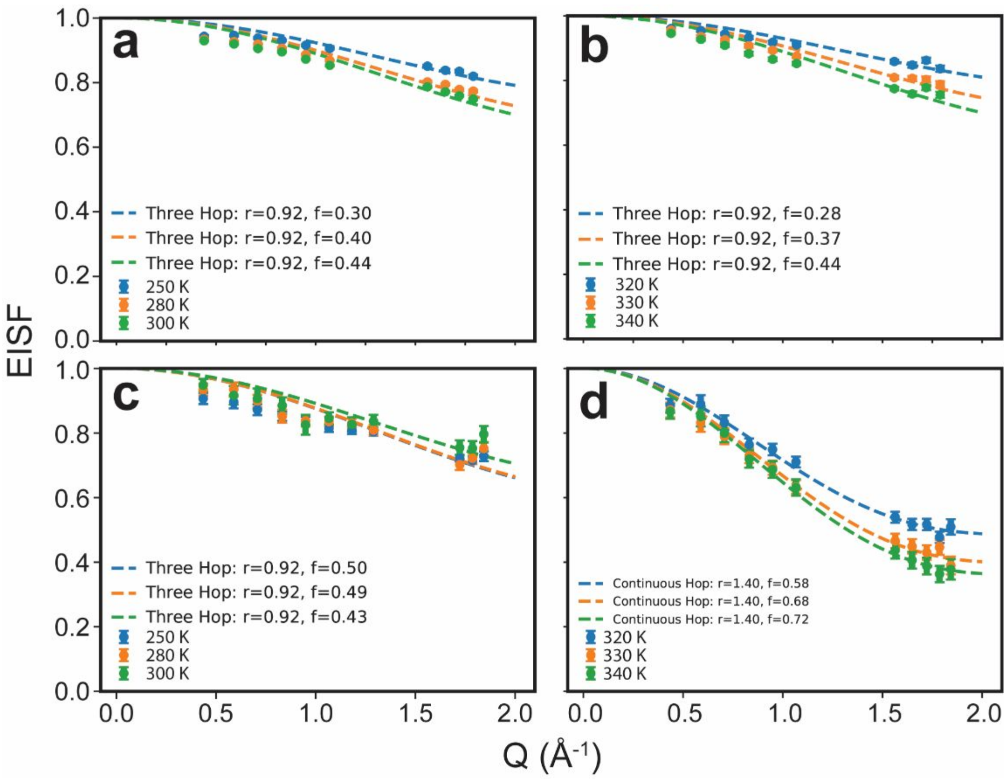

Figure S3 | EISF data and best fits. (**a**) Mode 1 below T_0 from IN5. (**b**) Mode 1 above T_0 from IN5. (**c**) Mode 1 below T_0 from IN16B. (**d**) Mode 2 above T_0 from IN16B.

Figure S3 provides the full EISF data for each of the observed modes, not shown in the main manuscript, on both IN5 and IN16B. Figure S3a,b gives the methyl group rotation (Mode 1) at all measured temperatures from IN5. Figure S3c provides the methyl group rotation (Mode 1) below the phase transition on IN16B and Fig. S3d gives the hydroxymethyl rotation (Mode 2) above the phase transition on IN16B.

Molecular Geometry Considerations

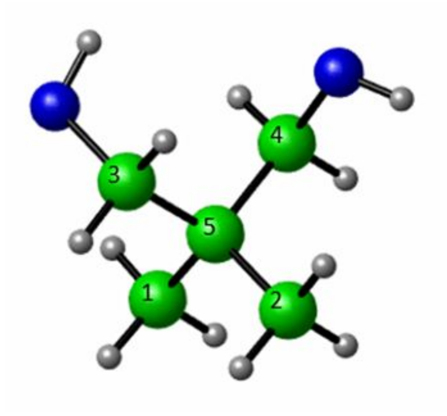

Figure S4: *NPG structure with C illustrated in light green, O in blue and H in light grey. See text for discussion of numbering.*

Structural details were taken from Cambridge Crystallographic Database deposition 832035 (doi: 10.5517/ccwxsvd), which was collected at a temperature of 173 K. The NPG molecule is shown schematically in Fig. S4. It is worth noting that because the environment of the molecule in the crystal structure is not symmetric, there are small variations in bond angles and lengths that may not be apparent in the plastic crystal phase. For example, for the hydroxymethyl groups, one O-C-C bond angle is 113. 8° whilst the other is 112.7°. This is not expected to be significant within the approximations of the QENS model used.

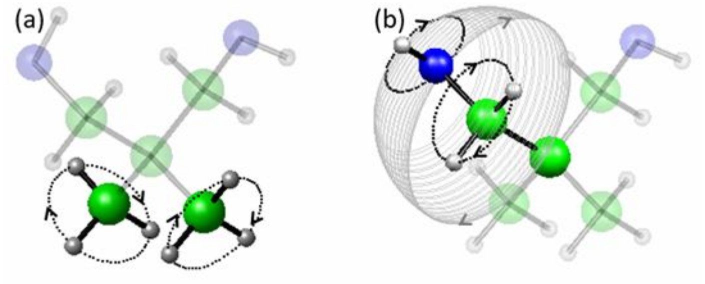

Figure S5: *Schematic showing rotation of the (a) methyl and (b) hydroxymethyl hydrogen atoms. The locus of hydroxyl hydrogen positions resembles a section of a spherical cap, illustrated in grey.*

Rotation of the methyl groups about the bond to the central carbon (the C1-C5 and C2-C5 bonds as illustrated) moves the methyl hydrogen atoms in circular orbits of radius of 0.92 Å, as shown in Fig. S5a.

Two of hydroxymethyl carbons, such as those bonded to C3 in Fig. S4, rotate about the bond to the central carbon (eg. the C3-C5 bond) with a similar radius to that of the methyl hydrogens (0.94Å). The hydroxyl hydrogen rotates about the O-C3 bond, which itself rotates about the C3-C5 bond, producing a range of motion resembling a section of a spherical cap, with the C3-C5 bond as its axis: see Fig. S5b. The average radius of rotation about the CC bond axis for the hydroxyl hydrogen atoms is 1.66 Å, giving an average for the three hydroxymethyl carbons of 1.18 Å.

Next, we consider the average distance of hydrogen atoms from the molecular centre of mass in order to estimate the average radius of rotation if the entire molecule is free to rotate in a tumbling motion. A MonteCarlo approach was taken, averaging the hydrogen positions with respect to the molecular centre of mass for 10^6 random molecular configurations, each set with randomly-assigned rotation angles for each rotational degree of freedom. The resulting distribution of radii is shown in Fig. S6 and yields an average hydrogen rotation radius of 2.23 Å.

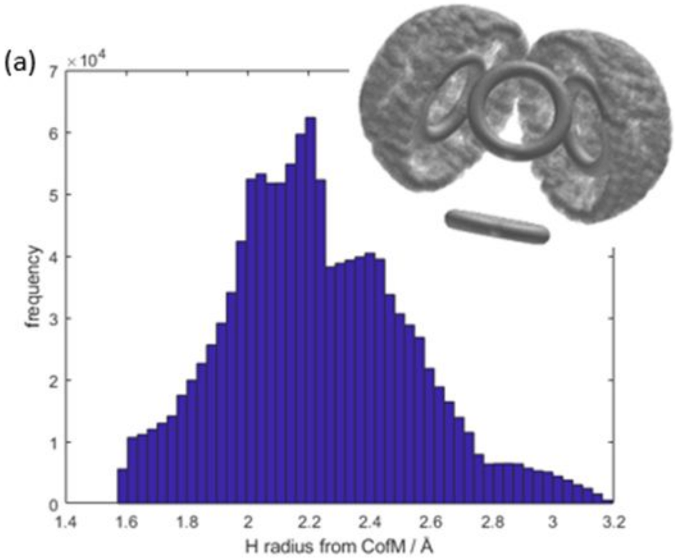

Figure S6: *(a) Distribution of hydrogen distances from the centre of mass of a tumbling NPG molecule with (inset) a representation of the possible hydrogen positions, where methyl hydrogens are restricted to circular motion and hydroxyl hydrogens move across a section of a spherical cap.*

QENS Global Fitting

Figure S7 shows the global fitting of QENS and FWS data from IN16B at low (0.2927 Å) and high (1.8426 Å) *Q* values.

We additionally explored combining IN5 and IN16B QENS datasets in a global fit across instruments, however we found that this gave the same results as shown in the main manuscript, when treating the instruments individual, because:

1. Below T_0, IN5 and IN16B only detect a single mode (CH_3 rotation) that agrees very well between both instruments. We found that we can obtain a more robust measurement of the activation energy of the CH_3 rotation by fitting the IN16B FWS data than by treating the combined IN16B and IN5 QENS data with an Arrhenius law (due to the small temperature separation of the QENS data points; 250 K, 280 K 300 K).
2. Above T_0, Modes 2 and 3 (observed only on IN16B) have linewidths ($\Gamma_2 \approx 20$ μeV and $\Gamma_3 \approx 3$ μeV) that are less than the FWHM energy resolution of IN5 (60 μev). As such, identification of these modes was unchanged when combining IN5 and IN16B datasets for a single fit. Likewise, Mode 1 (observed on IN5) had a FWHM linewidth of $\Gamma_1 \approx 50$ μeV, which is approximately equal to the energy range of IN16B (± 28 μev). As such, the fitting of this mode on IN16B was well described by the flat background term that was already included in the fit.

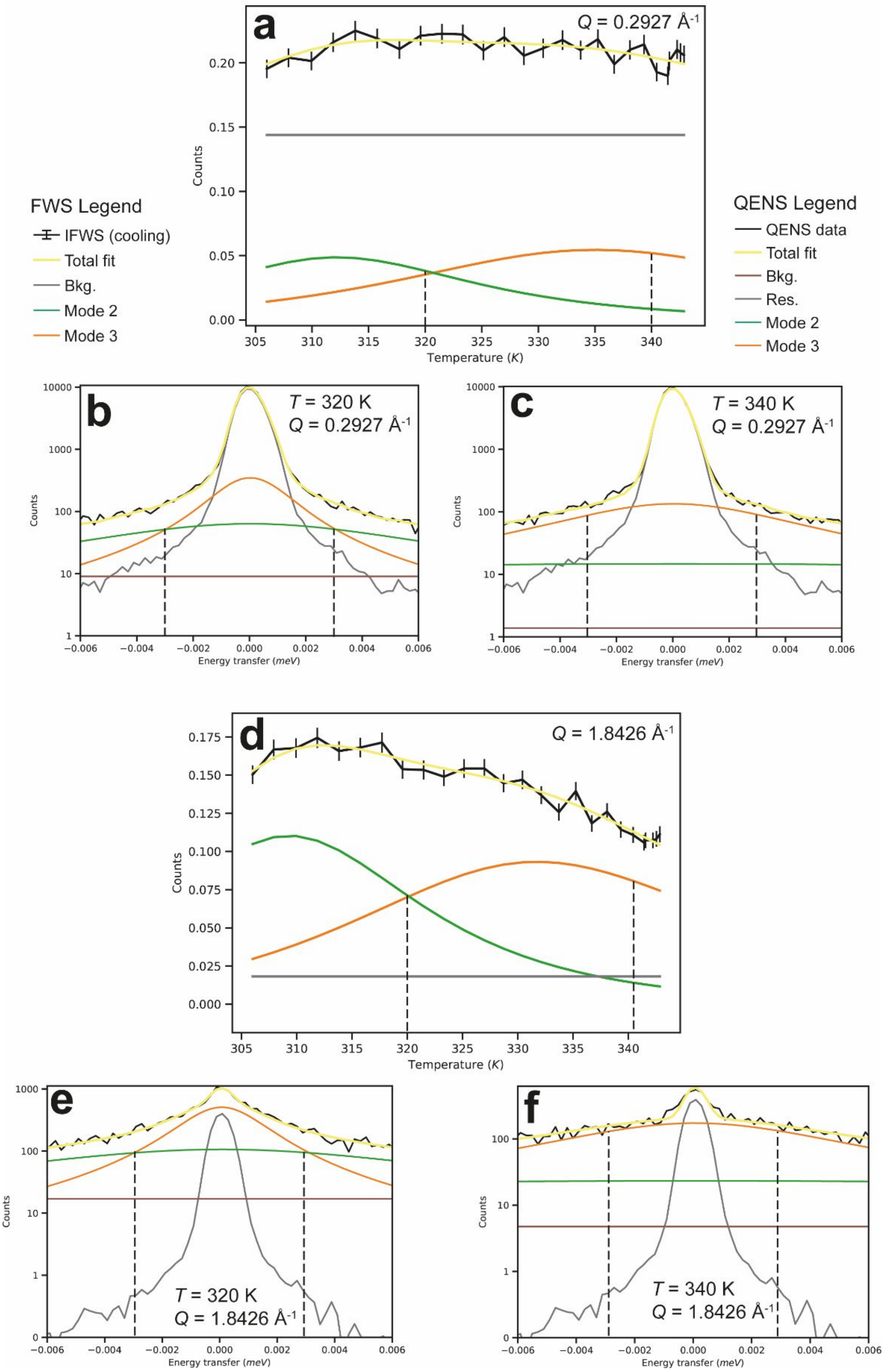

Figure S7 | FWS and QENS Global fits at low and high *Q***.** (**a**) IFWS global fit for *Q* = 0.2927 $Å^{-1}$. (b,c) QENS global fits for *Q* = 0.2927 $Å^{-1}$ at 320 K and 340 K respectively. (d) IFWS global fit for *Q* = 1.8426 $Å^{-1}$. (e,f) QENS global fits for *Q* = 1.8426 $Å^{-1}$ at 320 K and 340 K respectively. Dotted vertical lines in (a,d) indicate the temperature of the QENS scans. Dotted vertical lines in (b,c,e,f) indicate the energy offset for the FWS measurements (± 3 μeV).

(1) Rendell-Bhatti, F.; Boldrin, D.; Dilshad, M.; Moya, X.; MacLaren, D. A. Understanding Variations of Thermal Hysteresis in Barocaloric Plastic Crystal Neopentyl Glycol Using Correlative Microscopy and Calorimetry. *Journal of Physics: Energy* **2024**, *6* (2), 025020. https://doi.org/10.1088/2515-7655/AD3985.

(2) Lunkenheimer, P.; Loidl, A. 'Boson Peak'and High-Frequency Excitations in Glassy Crystals. *arXiv preprint cond-mat/0210067* **2002**, 1–4.

(3) Brand, R.; Lunkenheimer, P.; Loidl, A. Relaxation Dynamics in Plastic Crystals. *Journal of Chemical Physics* **2002**, *116* (23), 10386–10401. https://doi.org/10.1063/1.1477186.

(4) Salzillo, T.; Girlando, A.; Brillante, A. Revisiting the Disorder-Order Transition in 1-X-Adamantane Plastic Crystals: Rayleigh Wing, Boson Peak, and Lattice Phonons. *Journal of Physical Chemistry C* **2021**, *125* (13), 7384–7391. https://doi.org/10.1021/acs.jpcc.1c00239.
